# Barriers and facilitators for treatment and control of high blood pressure among hypertensive patients in Kathmandu, Nepal: a qualitative study informed by COM-B model of behavior change

**DOI:** 10.1186/s12889-021-11548-4

**Published:** 2021-08-09

**Authors:** Buna Bhandari, Padmanesan Narasimhan, Abhinav Vaidya, Madhusudan Subedi, Rohan Jayasuriya

**Affiliations:** 1 School of Population Health, The Faculty of Medicine and Health, The University of New South Wales, Sydney, NSW 2052 Australia; 2 Central Department of Public Health, Tribhuvan University Institute of Medicine, Maharajgunj, Kathmandu, Nepal; 3 Department of Community Medicine, Kathmandu Medical College and Teaching Hospital, Sinamangal, Kathmandu, Nepal; 4 School of Public Health, Patan Academy of Health Sciences, Lalitpur, Nepal

**Keywords:** Hypertension, Adherence, Stigma, Formative study, Lifestyle

## Abstract

**Background:**

Nepal has a high prevalence of hypertension which is a major risk factor for cardiovascular diseases globally. It is inadequately controlled even after its diagnosis despite the availability of effective treatment of hypertension. There is a need for an in-depth understanding of the barriers and facilitators using theory to inform interventions to improve the control of hypertension. This formative study was conducted to address this gap by exploring the perceived facilitators and barriers to treatment and control of hypertension in Nepal.

**Methods:**

We conducted in-depth interviews (IDIs) among hypertensive patients, their family members, healthcare providers and key informants at primary (health posts and primary health care center) and tertiary level (Kathmandu Medical College) facilities in Kathmandu, Nepal. Additionally, data were collected using focus group discussions (FGDs) with hypertensive patients. Recordings of IDIs and FGDs were transcribed, coded both inductively and deductively, and subthemes generated. The emerging subthemes were mapped to the Capability, Opportunity, and Motivation-Behaviour (COM-B) model using a deductive approach.

**Results:**

Major uncovered themes as capability barriers were misconceptions about hypertension, its treatment and difficulties in modifying behaviour. Faith in alternative medicine and fear of the consequences of established treatment were identified as motivation barriers. A lack of communication between patients and providers, stigma related to hypertension and fear of its disclosure, and socio-cultural factors shaping health behaviour were identified as opportunity barriers in the COM-B model. The perceived threat of the disease, a reflective motivator, was a facilitator in adhering to treatment.

**Conclusions:**

This formative study, using the COM-B model of behaviour change identified several known and unknown barriers and facilitators that influence poor control of blood pressure among people diagnosed with hypertension in Kathmandu, Nepal. These findings need to be considered when developing targeted interventions to improve treatment adherence and blood pressure control of hypertensive patients.

**Supplementary Information:**

The online version contains supplementary material available at 10.1186/s12889-021-11548-4.

## Background

Hypertension is a major risk factor for cardiovascular disease (CVD) [1]. It is reported to be a leading contributor to premature death and disability in the Global Burden of Diseases study in 2017 [2]. The global Disability-Adjusted Life Years (DALYs) due to hypertension has increased by 40% from 1990 to 2016 [3]. In low and middle-income countries (LMICs), the prevalence of hypertension is reported to have increased by 7.7% between 2000 and 2010 [4]. In a 2014 study, 27% of participants in the South Asian Association for Regional Cooperation (SAARC) region were found to have hypertension [5]. Nepal has witnessed an increment in hypertension from 21.5% [6] in 2008 to 24.5% [7] in 2019, as reported in the STEP Wise Approach to Surveillance (STEPS) surveys.

Despite clear evidence regarding the effective treatment of blood pressure using medication, global studies have reported that around half of diagnosed hypertensive patients remain untreated and more than half of those being treated continue to have uncontrolled blood pressure [8]. Studies in Nepal have found unacceptably high rates of uncontrolled blood pressure including 67% of the patients under treatment in a hospital-based study [9]; 65% in a community-based study of Central Nepal [10]; and 85% in diagnosed patients in a community-based study of Western Nepal [11].

Systematic reviews on barriers to management of high blood pressure have reported factors related to patients, providers and the health system [12, 13]. Several reviews have identified factors related to medication adherence for hypertension [14–16]. Recent studies conducted in Nepal also have shown inadequate knowledge of hypertension and its treatment, poor medication adherence, irregular follow-up, lack of availability of uniform treatment guidelines, and the inability of healthcare providers to deliver lifestyle modification messages as major challenges for high blood pressure management [17, 18]. However, there are no clear insights into the underlying factors of treatment adherence and blood pressure control to facilitate the design of appropriate interventions. There is an evidence that supports the use of behavioural theory in designing health interventions for effective outcomes [19]. Therefore, this formative study was conducted using a well-accepted theory, the COM-B Model [20] for behaviour change to not only identify and analyse facilitators and barriers to treatment and control of high blood pressure among hypertensive patients, but also to inform the development of behaviour change interventions.

## Methods

### Study setting

Nepal is a Low Income Country (LIC) where 25.2% of people are living below the poverty line [21]. In Nepal, out-of-pocket healthcare expenditure was 57.8% of the share of the total healthcare expenditure in 2017 [22]. Primary care is delivered through a network of health posts (HPs), and primary healthcare centres (PHCCs) which are the first points of contact with the health system. Patients suffering from non-communicable chronic diseases are usually referred to hospitals with specialist facilities generally located in urban areas. We initially selected HPs and PHCCs of the Kageswori Manahara Municipality of Kathmandu, a semi-urban area. However, we found that hypertensive patients were visiting Central tertiary level healthcare facilities to receive treatment. Hence, this study was also conducted at Kathmandu Medical College and Teaching Hospital (KMCTH) to obtain a comprehensive picture of barriers and facilitators faced by hypertensive patients at different levels of the health system. KMCTH is a tertiary level referral health facility, where patients come from various parts of the country.

### Study methods

A qualitative study was conducted among patients and providers to explore their perspectives on barriers and facilitators to treatment and control among hypertensive patients in Nepal. Qualitative methods are appropriate for formative research where there is a need to explore determinant factors that are specific to a context and where literature is limited. Qualitative methods can provide deep insights into the problem being studied [23]. The findings of this formative study were used in informing the intervention (TEXT4BP), a pilot randomized control trial being tested among hypertensive patients in Nepal [24].

### Study participants

To obtain diverse views from all critical stakeholders, we included hypertensive patients, their family members, healthcare providers working at different levels of the health system and key informants. We included patients (ranging from 30 to 70 years old) who had been diagnosed with hypertension and were prescribed anti-hypertensive drugs by a qualified medical practitioner. To ensure maximum variability, we undertook purposive sampling of patients with diverse characteristics (age, sex, level of education, duration of disease and status of control). We excluded hypertensive patients with serious complications (e.g., stroke, myocardial infarction, kidney diseases etc.) and those with co-morbidities requiring advanced care at a secondary or higher-level hospital as these limited their ability to participate in the study. However, we included patients with co-morbidities who were not serious and did not require immediate treatment. Patients with severe mental and physical disabilities and pregnant women were also excluded. Healthcare workers from PHCCs and HPs of Kageswori Manahara Municipality (primary level) and KMCTH (tertiary level) were interviewed.

### Recruitment

Healthcare workers distributed a study flyer containing information on the objectives, eligibility, and nature of the study to eligible hypertensive patients at selected health facilities. A list of those who agreed to participate was provided to researchers along with participant contact details. Participants, of both FGDs and IDIs, were asked to nominate and obtain consent from those family members who were involved in their care so that the researchers could contact family members purposively. The healthcare providers from selected sites were also invited for an interview to obtain their perspectives. To gather policy-level perspectives on challenges faced by the patients, key informants working in the Non-Communicable Diseases (NCDs) management (policy makers, researcher, NCDs program focal persons at Department of Health services, MoHP) were interviewed in the study. Details are presented in the Tables 1 and 2.

**Table 1:** Sociodemographic profile of the study participants (Hypertensive patients)

Characteristics	Categories	Hypertensive patients IDIs (***N*** = 25) (%)	Hypertensive patients FGDs (***N*** = 16) (%)
Age (years)	30–40	6 (24)	4 (25)
	40–50	5 (20)	6 (38)
	50–60	7 (28)	5 (31)
	60–70	7 (28)	1 (6)
	Mean age ± SD	50.2 ± 11.3	44.93 ± 10.9
Sex	Female	14 (56)	7 (44)
	Male	11 (44)	9 (56)
Education	Literate	16 (64)	11 (69)
	Illiterate	9 (36)	5 (31)
Occupation	Employed	15 (60)	14 (87)
	Unemployed	10 (40)	2 (13)
Blood pressure status	Controlled (< 140/90)	15 (60)	9 (56)
	Uncontrolled (≥140/90)	10 (40)	7 (44)
Duration of diagnosis of hypertension	Less than a year	2 (8)	3 (19)
	1–5 years	12 (48)	10 (62)
	More than 5 years	11 (44)	3 (19)

**Table 2:** Characteristics Health care providers, Key informants, and family members of hypertensive patients included in the study

Characteristics of health care providers (***N*** = 11)		Characteristics of key informants (***N*** = 4)	Characteristics of family members (***N*** = 5)	
**Sex**				
Female	4	1	Female	3
Male	7	3	Male	2
**Position**			**Education**	
Cardiologist	3	NCD Policy maker −1	Literate	3
Physician and medical officer	3	NCD program focal person at DoHS, Ministry of health – 2	Illiterate	2
Health Assistant and Community Medical Assistant	3	NCD Researcher - 1	**Relationship with hypertensive patients**	
Nurse (ANM, Staff nurse)	2		Wife	1
**Work experience**			Husband	1
1–5 years	4	1	Daughter	1
5–10 years	4	1	Son	1
More than 10 years	3	2	Daughter in law	1
**Level of health care**				
Primary level	5	Both level - 4	Primary level	3
Tertiary level	6		Tertiary level	2

### The behavioural model informing the study

Barriers to high blood pressure treatment and control were defined as any factors restricting the performance of recommended behaviours for patients to maintain optimum blood pressure control and treatment (medication and lifestyle) [25]. The COM-B model a component of the Behaviour Change Wheel (BCW), developed by Michie et al. [20, 26] was used as the overarching model in this study. The study utilized this model to understand the capability, motivation and opportunity barriers and facilitators for treatment and control of high blood pressure faced by patients. In addition, providers perspective was sought on the relevant components of the COM-B Model, for instance, opportunity barriers faced by patients in seeking and maintenance of treatment. This model has been used in similar studies in other settings [27–30] to understand the perspectives of both the service users and providers [31–33].

**Fig. 1:**
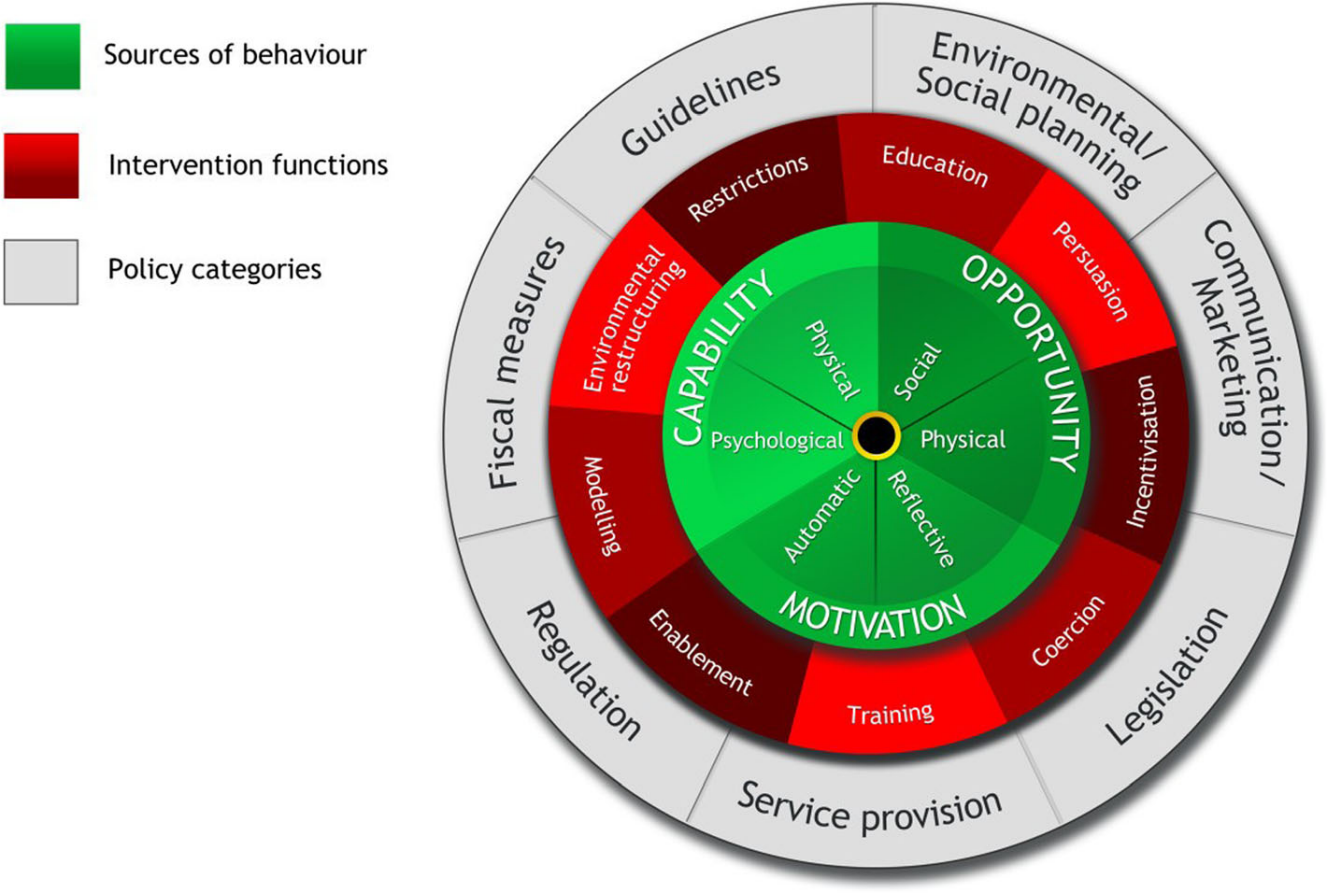
The Behaviour Change Wheel [20, 26] ^[published with permission from Michie S]^

The COM-B model (Fig. 1) involves three interacting components: Capability, Opportunity and Motivation. These components are further divided into six sub-components [20, 26]. Capability comprises psychological and physical capability. Psychological Capability is the capacity to engage in necessary thought processes, while physical capability encompasses the capacity of any individual to engage in a necessary physical process to modify their lifestyle and adapt to change [20]. The component opportunity refers to the external factors of the individual that makes behaviour change possible or prompts change, comprising two components–physical opportunity and social opportunity. Physical opportunity is provided by the environment; Social opportunity is the cultural milieu and social norms which dictates the way an individual thinks about things [20]. Motivation refers to “all the brain processes that energize and direct behaviour, not just one’s goals and conscious decision making” [20] and includes habitual processes, emotional responses, and analytical decision-making. There are two components of motivation in the COM-B model: reflective motivation and automatic motivation. The former involves evaluations and plans; the latter deals with emotions and impulses [20]. We used the COM-B model in developing interview guides to elicit barriers and facilitators during data collection. During analysis, the COM-B components were used as a framework to categorise the emerging sub-themes during thematic analysis.

### Data collection

We used in-depth interviews (IDIs) and focus group discussions (FGDs) methods to collect data from March to July 2018. All IDI and FGD topic guides were translated into the Nepali language and were pilot tested (Additional file 1: IDI and FGD guides). We conducted 25 IDIs among hypertensive patients (13 primary level, 12 tertiary level), five IDIs among family members, 11 IDIs among healthcare workers (five primary level, six tertiary level) and four IDIs among key informants. There were no refusals to participate in the IDIs and FGDs. The first author (BB, PhD fellow), a native female health professional with over 10 years’ experience in NCDs as a faculty and researcher trained in qualitative methods, conducted all interviews at selected venues that provided privacy for participants (private rooms of OPD of the selected health centres and at the homes of the participants at the primary care level). An audio-recorder was used to record the interviews. Field notes were taken by the interviewer and used during the analysis to understand the context and nuances of the responses. IDIs took 35 to 60 min each. Repeat interviews were not required. Two FGDs (8 patients in each) were conducted among 16 hypertensive patients who represented a diverse group of participants (Tertiary level). The first author (BB) facilitated the FGDs in the Nepali language. Each FGD lasted 60 min to 90 min. At both venues there were no additional people present apart from the interviewer during IDIs and FGDs. The number of participants for FGDs was based on the standard recommendations for FGDs [34] and patient-availability. The number of IDIs for patients was determined to obtain wide diversity by age, sex, level of education, duration of disease and controlled/uncontrolled blood pressure. Similarly, diversity was sought among the providers. The numbers were limited by reaching saturation in new findings. After each interview, the interviewer prepared the summary to see if any new ideas were emerging from IDIs to decide on the saturation of information.

### Data analysis

The first author (BB) transcribed 25% of the transcripts, and a qualified transcriber, fluent in both English and Nepali transcribed the remaining audio-recorded FGDs and IDIs. Before direct English transcription, three interviews were transcribed in Nepali and translated into English by the qualified transcriber. The English language transcripts were back translated by BB, and translation reliability and accuracy were compared with the original Nepali transcript. This process ensured the presence of no significant differences in translation. The transcripts were checked against audio recordings, and pseudonyms were applied to participants to maintain confidentiality.

QRS NVivo 12 software [35] was used for data management and coding using a codebook. Thematic analysis process outlined by Braun and Clarke [36] was used to analyse the data which involved searching, coding and categorizing words by words, lines by lines, and codes by codes throughout the dataset to identify a repetitive pattern of themes [36].

All transcriptions were coded independently by the first author (BB). A random sample of three transcripts was shared with a co-author (RJ) for independent coding verification. Any discrepancies were resolved and agreed upon between investigators. We have used different methods of data collection like IDIs and FGDs and included the perspectives of diverse groups (patients, their family members, healthcare providers and key informants) in the study. Different methods and sources of data complemented the findings of each other. The initial codes generated from inductive coding were merged into subthemes and those emerging subthemes were mapped under the COM-B model using a deductive approach. For example, the codes that emerged inductively (“Fear of teasing/exclusion by friends; taking medicine secretly; not disclosing reasons for low salt diet; marriage prospects low on disclosure”) generated the subtheme “Stigma of disease and fear of disclosure”. We then mapped out these subthemes to themes based on the COM-B model using a deductive approach. The Consolidated Criteria for Reporting Qualitative Studies (COREQ) 32-item checklist [37] was followed to ensure reporting consistency.

## Results

The characteristics of hypertensive patients who participated in IDIs and FGDs are presented in Table 1**.** The mean age of participants in IDIs was 50.2 years and in FGDs was 44.9 years. There were 44% males in IDIs and 56% in FGDs; 64 and 69% of patients were literate in IDIs and FGDs, respectively. The distribution of patients with blood pressure control was 60% in IDIs and 56% in FGDs.

Detail characteristics of other participants (HCWs, Key informants, family members) are provided in Table 2.

The themes and sub themes derived from the study are presented in Table 3.

**Table 3:** Mapping of themes to the COM-B Model

Emerging sub-theme from the transcript	Sub-components of the COM-B Model	Broad components of COM-B Model
Poor knowledge and understanding about the disease and treatment (B)	Psychological Capability	Capability
Misconceptions about the disease and treatment (B)		
Physical challenges in taking treatment and modifying behaviour(B)	Physical Capability	
Affordability: Cost of the hospital, medicine, investigation, travel costs (B)	Physical Opportunity	Opportunity
Availability: Hospitals, medicine, investigation, chemist shop (B)		
Communication between providers and patients (B)		
Stigma of disease and fear of disclosure (B)	Social Opportunity	
Socio-cultural beliefs shaping health behaviours (B)		
Beliefs about consequences of disease and treatment (B,F)	Reflective Motivation	Motivation
Belief and trust in alternative medicine (B)		
Difficulty in changing habits (B)	Automatic Motivation	

B *barriers*, *F* facilitators

### Capability

#### Psychological capability

Sub themes under psychological capability comprised of:

##### Poor knowledge and understanding of disease and treatment

Responses showed that many patients lacked necessary knowledge and understanding of various causes and risks associated with hypertension. Some were not aware of symptoms of hypertension and that it can be an asymptomatic disease. As one participant described:

*“If it is increased … what will happen... like... [Inaudible]... if increased may be unconscious or fall … may faint … I don’t know what happens.” (P08:50-55Y, PHC level).*

Many study participants did not have adequate knowledge about the complications of hypertension and were unaware of the consequences of uncontrolled blood pressure. They did express the need for more information about risk factors and treatment of hypertension.

Many were not aware of recommended behaviour modifications for blood pressure control. They did share their knowledge regarding the significance of exercise and salt reduction in their diet to control blood pressure; however, they did not know about the kind of exercises required for this and expressed their concern about salt intake. For example,

*“Another thing is ... neither I know what exactly salt will do or how it will affect in my pressure... I don’t have exact information.” (FGD1- P02: 40-45Y, Tertiary level).*

Many study participants reported the misinformation that adding water to their diet would reduce the amount of salt in it. On the other hand, if they were advised to reduce salt, the health providers were worried that they would restrict salt to such an extent that there would be other issues like hyponatraemia. As one health provider explained:

*“Yes, they are cooking food without salt and taking out vegetables for them, and they will add salt for the family members … They are not taking salt completely, and another problem will arise.” (HCW 01, PHC level).*

Some were aware that heart attack and sudden death could be a result of uncontrolled blood pressure.

#### Misconceptions about the disease and its treatment

Participants revealed many prevailing misconceptions related to the association between disease and age. Some stated that hypertension is a disease of old age, and therefore, medicine should not be started at a young age. They stated that if people get hypertension in their youth, they should delay the treatment until they reach fifty. One participant summed this approach up aptly:

*"Half of the people I met said that I am too young to take medicine for pressure. They said that I should not be taking medicine before fifty years of age." (P014: 30-35Y, Tertiary level)*

Patients considered anti-hypertensive medication as the last option after trying other alternatives. Hence, they were taking the anti-hypertensive medicine after getting complications and at late stage. Moreover, they were worried about taking medicine for a long time. Participants also expressed that they do not need to take medication until and unless they felt symptoms such as pain or discomfort. Healthcare providers agreed that patients usually do not start their treatment on time, and if started, it was after trying all other alternatives. Patients were likely to discontinue the treatment when their pressure was under control. As one participant described:

*"I went to see the doctor (A-pseudonym) … Doctor checked my pressure, and it was 170/100. He gave me medicine… I took the medicine for 3-4 days and my pressure was in control, and I stopped that medicine." (P020: 35-40Y, Tertiary level)*

It was evident that deeply rooted perceptions were guiding the hypertensive patients’ behaviour, either they were not taking treatment or stopping them in between and not following recommended behaviour.

#### Physical capability

During analysis, the following sub-themes related to physical capability were identified:

##### Physical challenges in medication adherence and lifestyle change

There were many barriers and challenges in taking the medication regularly. Among those related to physical capability were (i) forgetfulness, (ii) inconvenience, and (iii) being too busy with other commitments. Forgetfulness or memory was also reported as a reason for not taking medicine on time. Though forgetfulness is a psychological factor, it resulted in challenges to the physical act of taking medicine. This was reported not only by elderly participants but even by young participants. For instance, a young participant expressed the need for a reminder to take his medication: “*If there would be something to remind me, then that might be easy for me”*.

*"And yes, sometimes I forget to take medicine as well. Medicine which needs to be taken in the morning, sometimes I forget and take that in the evening. Sometimes, I don't remember that as well." (P021: 35-40Y, Tertiary level)*

It was evident that most patients faced challenges in changing their lifestyles. They reported a lack of skills and difficulty in managing time for exercise, giving exercise a low priority, negligence to take medicine and not following the recommended behaviour as the main challenges. One patient briefly explains:

*"For physical activity, it would be good If I walk in the morning, but I don't feel waking up from the bed in the morning." (P011: 60-65Y, PHC level)*

Mostly female participants explained that they could not manage their time for the required physical activity due to their household commitments. Differentially assigned roles based on patient sex might also have affected their ability to modify behaviour in the study setting.

### Opportunity: physical opportunity

#### Availability and affordability of the health services

Access to health services and affordability of the treatment were expressed as important system-level challenges. Given the shortcomings in access to healthcare services for most patients in Nepal, there were many issues that pertained to a lack of appropriate medicine, no clear guidelines, a lack of skilled health workers, and a lack of training on NCDs at the primary healthcare level. A major barrier to treatment and follow up is a lack of universal access to health services and the requirement to access care at the tertiary level. Costs of medicine and diagnostic tests, lack of health insurance and heavy workloads of doctors at the tertiary level resulted in many participants discontinuing their treatment or inconsistent follow up.

*"But here people in rural areas are not able to get proper treatment. There is less amount of health posts in those areas and with very little manpower. There is also a problem related to the availability of drugs. First is the geographical barrier; second is transportation problem; third is lack of health-related infrastructure; the fourth is lack of manpower and the fifth is the cost factor."* (KI 04)

In Nepal, anti-hypertensive medicine is not provided free of cost through the healthcare system. In PHC level, even participants expressed the concern of non-availability of antihypertensive medicine/pharmacy in nearby places.

*We cannot buy these medicine (antihypertensive) around here. We must go there (city) for buying it. We must go till Chabahil (name of city -2-3 hr travel).* *Yes,* *must go that much far for buying it. I usually buy for a month for my mother. (Son of P01, 60-65Y, PHC level)*

Medicine needs to be taken continuously for the patient’s life, so the issues of availability, cost of anti-hypertensive medicine and diagnostic tests were raised by the study participants. One of the key informants expressed that a “*country like ours haven’t [sic] got the insurance policy and patient must buy the medicine from their own money and affordability is also one major barrier*.”

##### Communication between providers and patients

Poor communication between providers and patients was one of the most important sub-themes that was elicited during IDIs. Many patients complained that their provider did not tell them about their diagnosis, medicine, and recommended behaviour modification. Some patients learned about their high blood pressure status after taking medication for almost 2 years.

*"Now … . in the hospital, they will not tell in detail … They say, "your pressure is high, so you have to take medicine and write the name of the drug directly without any explanation." (P04: 60-65Y, PHC level)*

Healthcare providers admitted their shortcomings in counselling to make the patient understand their disease and treatment. They stated that they could not give enough time to patients for counselling, due to heavy workloads.

"*One is the patient-doctor ratio. Especially in the government sector, there will be much rush. Must give plenty of time but we are not able to give that time." (HCW 010, Tertiary Level)*

Key informants also expressed that *“if the counselling is not done properly, they won’t start taking medicine.” (KI 02).*

Good communication plays a crucial role in enhancing the good relationship between the healthcare provider and patients. It helps patients to open-up about their problems, remove their doubts and enhance their trust in treatments.

##### *Social opportunity*

Is related to those cultural factors which determine a person’s behaviour [20]. The sub-themes of social opportunity that directly affect a patient’s behaviour are discussed below in detail:

#### The stigma of disease and fear of disclosure

Participants expressed fear to disclose their disease due to the social stigma attached to hypertension in their vicinity. For example, people with hypertension are considered generally weak and unable to perform their duties properly. Moreover, young, and unmarried male participants shared that it would be difficult for them to find a bride if they disclose their hypertension status. Two patients have expressed this stigma in detail:

*"But I feel like … after knowing this my friend might tease me about this. Maybe there will be a difference in their behaviour regarding taking food, and there might be some limitation. I cannot tell other people... I feel ashamed." (P021: 35-40Y, Tertiary level)*

*"They think that if they marry a person who has high pressure, they will be barriers to their marriage … Yes, people try to hide it also. They never say they have it even though they have pressure." (HCW 05, PHC level)*

Stigma attached to hypertension, may have influenced health seeking behaviours, and follow up of treatment leading to uncontrolled blood pressure and complications. This was more evident with younger participants.

#### Socio-cultural norms shaping health behaviour

In some ethnic groups in Nepal, alcohol is associated with *good luck (Sagun),* as an offering to God during worship, and is also consumed on special occasions. Such cultural norms encourage alcohol consumption. A healthcare worker describes this cultural practice:

*"In some community, it is like in their Sanskritic (cultural) program; alcohol is like necessary item. Drinks and alcohol should be there. Because of that, they are regularly consuming alcohol." (HCW 011, Tertiary Level)*

Furthermore, oily, and fatty foods are considered to be superior and delicious items and are commonly provided in feasts and festivals and offered to guests as a mark of hospitality. Consumption of salt also holds cultural importance. When there is a death of a close family member, people restrict salt in their diet to express their grief.

"*We have deeply rooted old superstition and tradition as if anyone left salt, they ...people will say ...look she is unfortunate (aalacchini ...) look she is not taking salt as if there is death in the family.* *aalacchini … look … . who died in your family, so you are not taking salt...? people will say like this." (P05: 50-55Y, PHC level)*

The tradition and practice of salt intake during grief was a new insight for the researcher (BB) who is a member of the same society with the same cultural understandings of the process of death. Traditional and cultural norms associate obesity with good luck and wealth. These deeply engrained norms may discourage hypertensive patients from losing weight and negatively affect blood pressure control.

#### Motivation

In this study, two sub-themes of reflective motivation were identified.

#### Beliefs regarding consequences of disease and treatment

Beliefs about the consequences of disease were identified as a motivator for behaviour modification, especially in taking anti-hypertensive medicine. Several participants admitted that they were taking medication or following the recommended behaviour due to a fear of complications. For instance, participants thought that they might have a heart attack or suffer paralysis if they stopped the treatments prescribed by doctors.

*“I am taking medicine. If I left 1-2 days, then I get afraid that I might get paralysis. (Laugh)... … I get scared of dying … and if I don't take medicine, then I can’t sleep as well.” (FGD2-06: 30-35Y, Tertiary level)*

They feared consequences of complications of the disease. They were scared that if they were paralyzed, their social life would be disturbed, they will be a burden to the family and may not get enough care. Participants also expressed fears of side effects of the medication and developing addiction to the medicine due to prolonged intake. Some hesitated to start taking the medication and discontinued after commencement. One participant describes this hesitation as:

*“I left completely.... it would become my addiction and habit … it would be like Nasha (addiction) so, I felt sad about... that I had to take it always, so I left that medicine.” (P021: 35-40Y, Tertiary level)*

As for treatment, some participants believed that missing a single dose of anti-hypertensive medication could lead to instant death. Therefore, they preferred uncontrolled blood pressure over starting the prescribed medication.

##### Belief and trust in alternative medicine

Participants stated that they had more faith in homoeopathy, ayurveda and other traditional herbs and bitter vegetables as remedies for high blood pressure as compared to allopathic (Western) medicine. Due to their strong trust in traditional medication, locally available remedies remained their first preference to Western medication. Some patients expressed their belief in the consumption of bitter substances as a supplement to cure or control of high blood pressure.

*“It’s been around one month that my pressure is little higher.... I used to drink the juice of bitter melon (karela) before … my children used to give me that. So, I did not take medicine.” (FGD1–07: 40-45Y, Tertiary level)*

The use of the juice of Jamara (green wheatgrass or barley sprouts) by Nepalese hypertensive patients was also reported during IDIs and FGDs. This is a recently introduced as a remedy for non-communicable diseases in Nepal. It also holds religious significance as it is used as a blessing during Dashain festival in Nepal.

“*While going to walk, he usually takes Jamara’s juice. That is only recently … and he also takes* *Aloe vera* *juice. It’s like if he heard anything like that will have a good impact on his health not only for the pressure; he usually takes that …” (wife of P014: 40-45Y, Tertiary level)*

These kinds of practices were favoured because they were understood to have less side effects, were easily accessible, were low cost, were advocated by close relatives and had religious significance.

#### Automatic motivation

##### Difficulty in changing habits

Participants also expressed behavioural inertia in changing lifestyles. For example, some were not following a recommended low salt and a low-fat diet. Participants also found it challenging to shift to a low-fat diet as oil was an integral part of their daily diet. In Nepalese society, a low salt and low-fat diet are considered to be diet for an ill person. These kinds of perspectives may shape their behaviour, choosing foods with high salt and fat to consider themselves healthy. Even family member admitted such challenges of changing in dietary practices.

*My wife (patient) loves eating fried meats and oily pickles (salty) a lot. I told her there is no use in having oily foods; control it. But it is the same. Nothing has changed. (Husband of P03: 35-40Y, PHC level)*

Similarly, participants confessed to being unsuccessful in trying to change their habits of smoking and consuming alcohol. An older participant residing in a rural area stated that she has been smoking since her childhood. Due to her long-term addiction, she was unsuccessful in her attempts to quit smoking even after being diagnosed with hypertension. She said, “*yes, I tried many times to stop smoking but could not.”*

*“I know everything... it will harm my pressure as well, but I am drinking (alcohol). It is like my challenge. I am taking medicine and taking alcohol, as well. It’s all due to my habit.”* (FGD2- 09: 55-60Y, *Tertiary Level)*

Hence, participant behaviours were guided by their entrenched lifestyles which are not easily modified**.**

## Discussion

We conducted a formative qualitative study guided by an evidence-based tool, the COM-B Model of Behaviour Change Wheel [20], to understand facilitators and barriers to the treatment and control of high blood pressure among hypertensive patients in Nepal and to inform our TEXT4BP intervention study [24]. This study used theory-based formative research to develop interventions for hypertension management in Nepal [24]. The study explored the knowledge and misconceptions of the disease and treatment, access to reliable information and health services and many social and cultural factors as the main barriers. Under-resourced clinicians, high costs, low health literacy and poor communication between patients and providers compounded issues relating to opportunity and equity of treatment. Perceived threats of disease were identified as the facilitators for behaviour modification. Importantly, this study has identified the stigma of the disease and fear of disclosure as one of the barriers to treatment and control of blood pressure, which is the unique finding of this study. The discussion that follows will look at the main barriers that can be targeted to design behavioural interventions in the study setting.

### Capability factors

Under the ‘capability’ theme, inadequate knowledge and various misconceptions about hypertension and its treatment were identified as barriers. These findings are consistent with previous studies conducted in Nepal [17, 18] and other similar LMICs [12, 38, 39]. In LMICs, illiteracy and inadequate access to information may significantly impact the knowledge and misconception of the disease and treatment. A lack of awareness and knowledge about hypertension may interfere with the timely diagnosis and management of blood pressure [12]. It plays a crucial role in modifying behaviour and seeking treatment which needs to be enhanced. The capabilities of patients with hypertension can be enhanced by providing education and skills training based on the BCW’s intervention functions [20]. The effectiveness of such educational interventions in improving knowledge about hypertension and treatment has been demonstrated by previous studies [40–42]. However, education alone might not be sufficient for the overall reduction of blood pressure [43].

Our study has identified forgetfulness and being too busy as barriers for medication adherence under the component of physical capability, where the former was reported even by younger patients. This may explain the findings of quantitative studies from other LMICs, where old age was associated with higher adherence [16]. Several systematic reviews [14, 16, 44] have discussed the poor medication adherence as one of the main barriers for hypertension control following diagnosis. Previous studies in Nepal have also documented low adherence to medication in the range of 35 to 64% [45, 46]. Many reviews have followed the five dimensions of the World Health Organization (WHO) Multidimensional Adherence model [47] to identify and classify barriers for medication adherence [14, 16, 44]. Our study found patient-related capability, motivation barriers, and provider-related opportunity barriers for medication adherence to be prominent of the five dimensions. Moreover, participants expressed their need for reminders to take medications. A study from three South Asian countries reported that family members reminder in taking medication improved medication adherence [48]. Reminders from the family, setting the alarm on mobile phones and using text messages have proven effective in improving adherence in previous studies in high-income countries (HICs) and in some LMICs [49, 50]. However, the feasibility and effectiveness of intervention using mobile technology in Nepal’s context is yet to be studied. This formative study findings were used to inform the design and contents of our planned mhealth intervention for the patients with hypertension in Nepal [24].

### Motivation factors

Additionally, a strong belief and trust in locally available herbs and bitter foods was identified as a barrier to the initiation and maintenance of allopathic medicines. Similar finding, where traditional medicines were preferred as a perceived cure for hypertension was reported in a study from Ghana [51]. A qualitative study from the Eastern part of Nepal also found the use of traditional medicines and herbs for the self-management of chronic obstructive pulmonary disease [52]. Participants might be more inclined to use those remedies due to local availability, encouragement by family members and beliefs that traditional medicines have no side effects and are low cost. There is no empirical evidence to support these beliefs in the efficacy of traditional remedies to control blood pressure to-date. These findings suggest that interventions need to address such beliefs in this setting. However, it is challenging to break that faith-thread shaped by culture. Further research can be done to investigate the efficacy of the holistic approach using alternative/complementary practices to control blood pressure.

The elicited theme ‘beliefs of consequences of disease and treatment,’ fits reflective motivation, another COM-B Model component. Participants identified fears of complications related to the disease as a prime motivator in starting medication and adhering to treatment. On the other hand, fear of the side effects and long-term usage leading to addiction resulted in either delaying initial treatment or stopping treatment shortly after commencement. Similar findings have been reported in Nepal [17, 18], Malaysia [53] and Colombia [38]. Fear of “dependence” on medication was reported in a systematic review [12] and other studies with a similar socioeconomic background [15, 54]. There is a need for adequate provision of information about disease and medication in the study setting.

### Opportunity factors

There were various physical opportunity barriers (related to healthcare systems and providers) elicited in our study. Previous reviews have often classified these under availability, affordability and accessibility barriers [12]. Similar accessibility barriers have also been reported in a multi-country study of rural areas of Pakistan, Bangladesh and Sri Lanka [48]. Such barriers can result in late diagnosis and irregular follow up. To address system-level barriers, equipping primary healthcare facilities for treatment of hypertension by strengthening capabilities of healthcare workers, development, and provision of contextual guidelines for hypertension treatment and adequate and timely supply of medicine should be reinforced. Recently, the government of Nepal piloted the Package of Essential Non-communicable Diseases (PEN) at health posts, primary healthcare centres and district hospitals of some districts for early detection and management of chronic diseases within the community [55], which might address some of these accessibility barriers. However, such program should be expanded to the whole country, and continuous monitoring/evaluation of the program is warranted to get the desired outcome.

The most significant barriers to behaviour change reported under the physical opportunity theme is “communication between providers and patients”. Thus, demanding workloads, a lack of time, a lack of counselling skills, and negligence were primary factors that resulted in poor doctor-patient relationships and led to mistrust in treatment and non-adherence to medicine. This finding is consistent with previous studies in Nepal [17, 18] and in other settings, as reported in a systematic review of qualitative and quantitative studies [12]. In the context of Nepal, where human resource shortage and work overload prevents required provider-patient communication, an alternative solution may be using mobile phone text messages to increase awareness and treatment adherence [50]. Training allied health staff or nurses in counselling [56] or involving family members [57] and peer groups in promoting self-care for hypertensive patients is another feasible option. There are tested strategies to improve provider communication at primary care levels in the west [58, 59], but these need to be adopted in Nepal.

Social influence was identified as both a barrier and facilitator for hypertension control in a systematic review [12]. Our analysis using the COM-B framework identified two themes (stigma of the disease and fear of disclosure, and socio-cultural beliefs shaping health behaviour) categorized under the component of social opportunity. Stigma was not elicited as a factor in previous studies in Nepal [17, 18]. However, some studies in Africa have reported that stigma was a barrier to treatment of hypertension [60]. Stigma has been documented as a barrier to care-seeking and treatment adherence for other diseases such as cancer [61]. Social opportunity factors such as social drinking as a cultural norm, obesity as a sign of wealth and low salt intake as bad luck were reported as major barriers to behaviour change. These were novel findings in our study compared to previous studies of Nepal [17, 18]. Many of these social influences require multifaceted interventions to change health behaviour guided by cultural norms, specifically, diets high in salt. A greater understanding of the causes and treatment of hypertension may reduce barriers due to stigma. Studies have used the health stigma and discrimination framework [62] in other settings to overcome barriers due to stigma.

### Implications for the policy and programs

All the barriers identified in the study are interrelated, so a holistic approach is required to address it at multiple levels such as patient, health system and community. It demands a policy-level action and commitment from the government and multiple other stakeholders such as civil society, academia, the private sector, and international organizations, in line with the recently endorsed Multi-sectoral Action Plan for the Prevention and Control of NCDs in Nepal (2014–2020) [63]. Primary health care facilities which are the first point of contact to the health system, should be well equipped for the management of NCDs such as hypertension. It requires training of primary HCWs, infrastructure development, adequate supply of anti-hypertensive medication, development, and implementation of uniform guidelines for diagnosis and treatment of hypertension. There is a need for culturally informed personalised education about hypertension and its treatment to the high-risk group at the individual and community level to address misconceptions and stigma in Nepal. Training of allied health workers, utilizing digital technology and using mass media such as radio and television for the information dissemination could be an alternative strategy to address the issue of scarce human resources for counselling patients at both levels of health care in Nepal. Further study is recommended to develop the contextual strategy at the country’s geographically disadvantaged areas, which was out of the scope of our study.

### Strengths and limitations of the study

This study’s strength lies in the data collection and analysis, which was informed by a behavioural model. It is the first formative study on hypertension management in Nepal to our knowledge. The study included participants from different healthcare systems and providers, unlike similar previous studies in Nepal [17, 18]. Additionally, we ensured trustworthiness [64] of data using (i) Different methods of data collection (IDIs and FGDs) (ii) Including different cadres of participants (patients, their family members, healthcare providers and key informants) and (iii) Member checking by summarizing and clarifying information and getting the participants’ validation during interviews. Participant validation was obtained to ensure representation of the respondent’s perspective [65, 66]. However, we did not provide a transcript to participants for their validation.

All interviews were conducted by the first author (BB), a native researcher sharing a similar cultural and linguistic background to the participants**.** One limitation is, as the researcher was a professional working in a tertiary centre, some participants may have assumed that she was familiar with the issues being discussed. However, to overcome this bias, the researcher probed each question. The finding of the study is not representative as it was conducted in one part of the country. It might not reflect the perspectives of the participants from the geographically disadvantaged parts of Nepal who depend on available local health facilities.

## Conclusions

This formative study, using the COM-B model of behaviour change, allowed the confirmation of several known and unknown barriers and facilitators that influence poor medication adherence and lifestyle change for control of blood pressure among hypertensive patients in Kathmandu, Nepal. This included stigma attached to hypertension, misconceptions about lifestyle change, and perceptions that low salt intake is bad luck, and that obesity is a sign of wealth. The findings can be a reference for academics, researchers, and policymakers of related fields for planning intervention programs to address the identified issues and for further research for better management and control of blood pressure among hypertensive patients.

## Supplementary Information

**Additional file 1.** IDI and FGD guides. Description: It contains the IDI guides for patients with high blood pressure (I), health care workers (II) and FGD guide for the patients with high blood pressure (III).

## Data Availability

Data is available on request from the corresponding author.

## Abbreviations

BCTs: Behaviour change techniques
BCW: Behaviour change wheel
CVD: Cardiovascular disease
COM-B: Capability, opportunity, and motivation- behaviour
DALYs: Disability-adjusted life years
DoHS: Department of Health Services
FGDs: Focus group discussions
HCW: Health care workers
HICs: High Income Country
HPs: Health Post
IDIs: In-depth interviews
KI: Key informants
KMCTH: Kathmandu Medical College and Teaching Hospital
LMIC: Low and middle-income Countries
LIC: Low income country
MOHP: Ministry of health and population
NCDs: Non-communicable diseases
P: Patients
PEN: The package of essential non-communicable diseases
PHC: Primary health care
PHCCs: Primary health care centres
STEPS: Stepwise approach to surveillance
WHO: World Health Organization
Y: Years
