# Supplementary material for: Barriers and facilitators for treatment and control of high blood pressure among hypertensive patients in Kathmandu, Nepal: a qualitative study informed by COM-B model of behavior change
Source: BMC Public Health. 2021 Aug 9;21:1524. doi: 10.1186/s12889-021-11548-4 (PMC8351340; doi:10.1186/s12889-021-11548-4)
Supplement: Supplementary file 1 — Additional file 1. IDI and FGD guides. Description: It contains the IDI guides for patients with high blood pressure (I), health care workers (II) and FGD guide for the patients with high blood pressure (III). [file 12889_2021_11548_MOESM1_ESM.pdf]

Additional file 1: IDIs and FGD guide

I. In-depth Interview guide for patients with high blood pressure)

1. Icebreaker

- How are you doing?
- What kinds of work are you doing these days?

2. Can you tell me in detail of how you came to know that you have high blood pressure and what you have been doing about it since then? *Probe: who diagnosed, what medications were given, whether he/she sought other treatment.*

3. Now, can you tell me about any difficulties you faced in getting your treatment at [mention place]. *Probe on physical and social factors: access, availability of medicines, affordability, what did the health personnel advice.*

4. So, once you obtained your medicines, can you tell me how you ensure that you follow the instructions given? Please describe the difficulties you have faced. *Probe: regularity, timing, preventing factors*

5. Can you please tell me who supports you to take your treatment at home and how they support you? *Probe: family support, friends or any other support*

As you know, there are other factors that might affect your high blood pressure

6. Could you please tell me what do you know about other factors that will increase or decrease your blood pressure? Probe: *[If they do not start – prompt by taking one example-] lifestyle factors*

Probe for capability (Knowledge) of patients: factors that increase blood pressure [high salt and fat intake, and lack of physical activity, smoking, alcoho]

7. In your opinion, what changes in these are needed for improving blood pressure control?? *Probe: Taking an example that they say that needs to be changed and ask for e.g. so you say smoking habits has to be changed, can you describe to me what efforts you have taken to change smoking habits.*

8. As you mentioned these obstacles you faced, can you tell me how you have or tried to overcome them? *Motivation (lack of interest, lack of discipline, previous experience, memory), Capacity (intensifies feelings of personal insufficiency, self-efficacy, physical problems). Probe: Intention and plans made*

9. Now, I like to discuss with you how you get information about your condition or health in general? Can you tell me who you go to and whose opinions matter most regards to information about your illness or other health issues? *Probe: Health workers, family, friends, mass media.*

10. Let's summarise some of the key points from our discussion. Is there anything else?

11. Do you want to share anything which I missed to discuss?

II. IDI guide for health care workers

1. We would like to find out from you about what you think are difficulties that patients with high blood pressure face in obtaining treatment for their high blood pressure? Can you tell us with examples, what they face and your views of why they face these obstacles and difficulties?

   Probe: do you know of any possible ways they can overcome the difficulties for example, in obtaining regular treatment.

2. So, once patients with high blood pressure start taking their medication, what prevents them or what difficulties do, they face taking the medicines regularly? Please describe in detail the challenges they face. *Probe: forgetting, not taking the appropriate dosage etc*

3. In your understanding, can you please tell me, what are the supports that patient should have, to take their treatment at home? *Probe: family support, friends or any other support*

4. In your understanding, are there any difficulties patient faces in obtaining treatment at [mention place]. *Probe on physical and social factors: affordability of treatment, availability of health workers, and medicine, a busy schedule of health workers, patients and provider's communication*

As you know, other factors affect the blood pressure of the patients

5. Could you please tell me what additional information you provide to patients to manage their blood pressure other than treatment? Probe: *[If they do not start – prompt by taking one example-] lifestyle factors that increase blood pressure [high salt and fat intake, and lack of physical activity, smoking, alcoholism]*

6. In your understanding, what kinds of obstacles do patients face in following recommended lifestyle change?

   Probe: *Motivation (lack of interest, lack of discipline, previous experience, memory),Capacity (intensifies feelings of personal insufficiency, self-efficacy, physical problems).*

7. Can you tell me what advice you have provided to them to overcome these obstacles?

8. Now, I like to ask you, in your understanding, how do these patients obtain information about their condition or health in general? Can you tell me whose opinions matter most to patients regards to information about their illness or other health issues? *Probe: Health workers, family, friends, mass media.*

10. Let’s summarize some of the key points from our discussion. Is there anything else?

11. Do you want to share anything which I missed to discuss?

III. FGD guide for patients with high blood pressure

Let's start by going around the circle and having each person introduce her/himself.

(Members of the research team should also introduce themselves and describe each of their roles.)

1. Can you tell me in brief one by one about how you came to know that you have high blood pressure and what you have been doing about it since then? *Probe: who diagnosed, what medications were given, whether he/she sought other treatment.*
2. Now, can you tell me about any difficulties you faced in obtaining your treatment at a Primary Health Centre /Health post or from private practitioners. *Probe on physical and social factors: access, availability of medicines, affordability, what did the health personnel advice.*
3. So, once you obtain your medicines, can you describe the difficulties you have faced in taking your medications as instructed. *Probe: regularity, timing, preventing factors*

[notice persons who are not sharing views and probe them as well]

4. Can you please tell me who supports you to take your treatment at home and how they support you? *Probe: family support, friends or any other support*

As you know, other factors affect your high blood pressure

5. Could you please tell me what you know about other factors that will increase or decrease your blood pressure? Probe: *[If they do not start – prompt by taking one example-] lifestyle factors. Probe for capability (Knowledge) of patients: factors that increase blood pressure [high salt, high fat intake, and lack of physical activity, smoking, alcohol]*
6. In your opinion, what changes are needed in your behaviour for improving blood pressure control and what obstacles have you faced?
7. You have mentioned several obstacles. Now can we take them one by one and find out how you have tried to overcome them? *Motivation (lack of interest, lack of discipline, previous*

experience, memory), Capacity (intensifies feelings of personal insufficiency, self-efficacy, physical problems). Probe for Intention and plans made.

8. Now, I like to discuss with you how you get information about your condition or health in general? Can you tell me who you go to and whose opinions matter most regards to information about your illness or other health issues? *Probe: Health workers, family, friends, mass media.*
9. Let’s summarize some of the key points from our discussion. Is there anything else?
10. Do you want to share anything which I missed to discuss?
